# A Wavelet-Recalibrated Semi-Supervised Network for Infrared Small Target Detection Under Data Scarcity

**DOI:** 10.3390/s25185677

**Published:** 2025-09-11

**Authors:** Cheng Jiang, Jingwen Ma, Xinpeng Zhang, Chiming Tong, Zhongqi Ma, Yongshi Jie

**Affiliations:** 1 Beijing Institute of Space Mechanics & Electricity, Beijing 100094, China; cheng3515523@163.com (C.J.); tongchiming.1989@163.com (C.T.); mzq11171079@163.com (Z.M.); jie_yongshi@163.com (Y.J.); 2 Ministry of Education and the Key Laboratory of Computer Vision and System of Ministry of Education, School of Computer Science and Engineering, Tianjin University of Technology, Tianjin 300387, China; xpzhang@email.tjut.edu.cn

**Keywords:** infrared small target detection, wavelet transform, semi-supervised learning

## Abstract

Infrared small target detection has long faced significant challenges due to the extremely small size of targets, low contrast, and the scarcity of annotated data. To tackle these issues, we propose a wavelet-recalibrated semi-supervised network (WRSSNet) that integrates synthetic data augmentation, feature reconstruction, and semi-supervised learning, aiming to fully exploit the potential of unlabeled infrared images under limited supervision. We construct a dataset containing 843 visible-light small target images and employ an improved CycleGAN model to convert them into high-quality pseudo-infrared images, effectively expanding the scale of training data for infrared small target detection. In addition, we design a lightweight wavelet-enhanced channel recalibration and fusion (WECRF) module, which integrates wavelet decomposition with both channel and spatial attention mechanisms. This module enables adaptive reweighting and efficient fusion of multi-scale features, highlighting high-frequency details and weak target responses. Extensive experiments on two public infrared small target datasets, NUAA-SIRST and IRSTD-1K, demonstrate that WRSSNet achieves superior detection accuracy and lower false alarm rates compared to several state-of-the-art methods, while maintaining low computational complexity.

## 1. Introduction

Infrared small target detection seeks to identify objects that occupy only a few pixels, lack discernible texture or structural features, and exhibit very low signal-to-noise ratios in scenes with complex backgrounds and strong interference [1,2]. The minute scale of these targets renders accurate detection extremely challenging. In recent years, the rapid progress of deep learning has enabled convolutional neural-network–based approaches to achieve notable advances in feature extraction and semantic representation, thereby opening new avenues for tackling this long-standing problem [3]. The effectiveness of deep models, however, relies heavily on large, high-quality annotated datasets [4]. Constructing such datasets for infrared small target detection is hindered by several factors. First, acquiring real infrared imagery is costly. This is especially true when the datasets need to cover diverse environmental conditions such as weather variations, terrain differences, and multiple imaging altitudes. In many cases, data collection is further limited by security or privacy concerns. Second, small targets in infrared imagery are inherently difficult to identify. They often appear as weak signals, which makes precise annotation extremely challenging. Producing high-quality labels requires both accurate localization and significant manual effort [5]. Because of these two challenges, namely data acquisition and annotation, the quantity of training samples currently available for infrared small target detection remains limited. This limitation prevents deep models from achieving robust and generalisable performance in complex scenarios. Consequently, data scarcity has become a critical bottleneck that hinders further progress in deep-learning-based infrared small target detection.

To address the scarcity of annotated data in infrared small target detection, an increasing number of studies in recent years have focused on the generation of synthetic infrared images. Traditional physics-based modeling approaches require the detailed modeling of target characteristics and imaging mechanisms. Although the generated results have a certain degree of physical realism, the modeling process is complex, computationally expensive, and lacks adaptability. In contrast, image synthesis methods driven by generative adversarial networks (GANs) have become the mainstream approach due to their end-to-end training capability, data-driven nature, and high flexibility. GAN-based synthesis methods can be further divided into two categories: data generation and style transfer methods [6]. Data generation methods do not rely on accurate imaging models, thus avoiding the complexity of physical modeling. However, the quality of the generated images can be affected by training stability and model generalization, which may lead to a lack of controllability and realism. Style transfer methods learn cross-modal mappings that preserve the structure and semantic consistency of the original images. These methods offer better adaptability and generation stability by enabling cross-domain feature transfer. Based on these advantages, we adopt an improved CycleGAN [7] model to map visible images into pseudo-infrared images. This strategy effectively expands the diversity and scale of the training data.

However, relying solely on the generation of synthetic infrared images remains insufficient to fully address the challenge of the weak feature representation of small targets in complex backgrounds. Although existing multi-scale feature fusion methods have improved detection performance to some extent, they still suffer from notable limitations. For instance, AGPCNet [8] adopted a multi-scale feature weighted fusion structure, but it did not fully account for the redundancy and dependency among features at different scales during the fusion process. This can lead to unstable target responses. Models such as UIU-Net [9] introduced a U-shaped architecture and employed skip connections to transfer shallow detail information. However, they were less effective in preserving high-frequency textures and edge details, particularly in scenarios involving extremely small targets or cluttered backgrounds. A common issue among these methods is the lack of targeted modeling in the multi-scale fusion process. Their limited feature representation capacity prevents them from effectively highlighting weak response areas associated with small targets. To enhance multi-scale feature expression, we design a lightweight wavelet-enhanced channel recalibration and fusion (WECRF) module. This module integrates wavelet decomposition with attention mechanisms to strengthen the modeling of high-frequency details and salient regions. It also employs channel recalibration to achieve adaptive feature fusion, thereby improving detection accuracy while maintaining a low computational overhead. The main contributions of this paper are as follows:

- We build a dataset of 843 visible-light small target images. Using an improved CycleGAN model, we generate pseudo-infrared images to expand the training data.
- We propose a wavelet-enhanced channel recalibration and fusion (WECRF) module, which combines wavelet decomposition with channel and spatial attention. It enables efficient multi-scale feature reweighting and fusion, enhancing feature representation.
- We design a wavelet recalibration semi-supervised network (WRSSNet). By fully leveraging unlabeled data, the network effectively addresses the limitations in feature learning and the representation caused by the scarcity of labeled samples.
- Our method achieves better performance than existing approaches on two public infrared small target datasets.

## 2. Related Work

### 2.1. Infrared Small Target Detection Method

Infrared small target detection methods can be broadly categorized into two main types: model-based approaches [10,11] and data-driven approaches [12]. Model-based methods include techniques based on image filtering [13,14], human visual system (HVS) [15,16] modeling, and low-rank approximation [17,18]. These methods perform well in scenarios with salient targets and relatively simple backgrounds. However, they often suffer from high false alarm rates and low recall in complex environments. To overcome the limitations of hand-crafted features in traditional approaches, a growing number of deep learning-based infrared small target detection methods have been proposed in recent years [19]. Models based on U-Net and Transformer architectures have achieved promising results. UCFNet [20] integrated central difference convolution and fast Fourier convolution to enhance feature extraction capability. Zhao et al. [21] reformulated the detection task as an image-to-image translation problem and introduced an L2 loss to improve localization accuracy, while employing a GAN to learn the distribution of target features. IAANet [22] utilized a region proposal network (RPN) and a Transformer encoder to model coarse target regions and generate attention-aware features, enabling precise target localization and effective false alarm suppression.

### 2.2. Synthetic Data

Due to the high cost of acquiring infrared images and the involvement of sensitive information, constructing large-scale and high-quality annotated datasets for infrared small target detection remains extremely challenging [23]. In recent years, many studies have focused on using synthetic data to alleviate the shortage of training samples [24]. Synthetic methods can generally be divided into two categories: physics-based modeling methods [25,26] and methods based on generative adversarial networks (GANs) [27,28]. Physics-based modeling methods simulate the imaging process, such as infrared radiation transmission and sensor response, to generate near-realistic images. These methods provide strong physical consistency but involve high development costs and complex parameters, which limit their adaptability to diverse scenarios. As a result, GAN-based methods have gradually become the mainstream approach. GAN-based methods can be further categorized into data generation and style transfer methods. For infrared small target detection, style transfer methods offer significant advantages. They can preserve the original structure of an image while effectively transferring infrared-style characteristics. For example, IR-GAN [29] employed a UConvNeXt generator based on ConvNeXt, introduced gradient vector loss, and incorporated a multi-scale PatchGAN to improve the quality and detail of generated infrared images. CycleGAN [7] used a pair of generators and discriminators to achieve unsupervised style transfer and image domain transformation through adversarial loss and cycle consistency loss.

### 2.3. Multi-Scale Feature Modeling

Infrared small targets are typically small in size with weak local features and are highly sensitive to scale variations. Therefore, effective multi-scale feature modeling is crucial for achieving high detection performance [30]. In recent years, multi-scale modeling has become a key research direction for improving infrared small target detection. Traditional structures like the Feature Pyramid Network (FPN) [31] enhance perception across different scales by constructing multi-resolution feature layers and performing top-down fusion. However, the fixed fusion strategy limits adaptability and makes it difficult to handle redundant information across scales. To address this, many methods have been proposed to improve multi-scale feature fusion strategies and enhance the representation of weak targets. For example, DNANet [32] introduced a densely nested multi-scale feature interaction structure combined with a channel-spatial attention mechanism, which effectively enhanced information integration across scales and improved saliency modeling for small target regions. MSDA-Net [33] incorporated multi-directional feature perception, local relational modeling, and high-frequency directional injection to efficiently integrate multi-scale structures with directional information.

## 3. Methods

### 3.1. The Overall Structure

**Figure 1:**
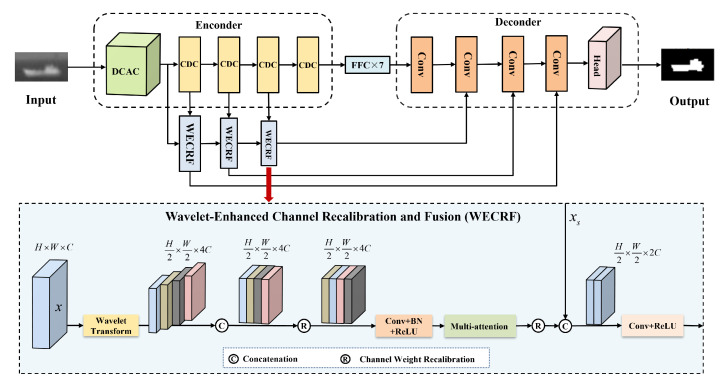
The architecture of WRSSNet. Three WECRF modules are used in the skip connections between the encoder and decoder to enhance the model’s multi-scale feature representation capability.

The proposed wavelet-recalibrated semi-supervised infrared small target detection method consists of two main stages. In the dataset construction stage, an improved CycleGAN model is employed to translate a constructed visible-light small target dataset into pseudo-infrared images, thereby augmenting the infrared data and enhancing the diversity of the training samples. Specifically, the generator and discriminator of the model are redesigned using a U-Net architecture, and a convolutional block attention module (CBAM) is integrated to strengthen the modeling of infrared modality features. It improves the realism and consistency of the generated pseudo-infrared images. In the target detection stage, a wavelet-enhanced channel recalibration and fusion (WECRF) module is proposed. It combines wavelet decomposition, channel attention, spatial attention, channel-wise recalibration, and feature fusion strategies to construct an efficient and lightweight feature aggregation mechanism. This module is incorporated into a wavelet recalibration semi-supervised network (WRSSNet), enabling fine-grained target segmentation and enhancing both the multi-scale representation of small targets and overall detection performance. Furthermore, the pseudo-infrared data generated in the dataset construction stage are utilized during training in the target detection stage, helping to expand the training set and alleviate the problem of insufficient labeled data. The structure of the WRSSNet model is illustrated in Figure 1.

### 3.2. Construction of a Visible-Light Small Target Dataset

**Figure 2:**
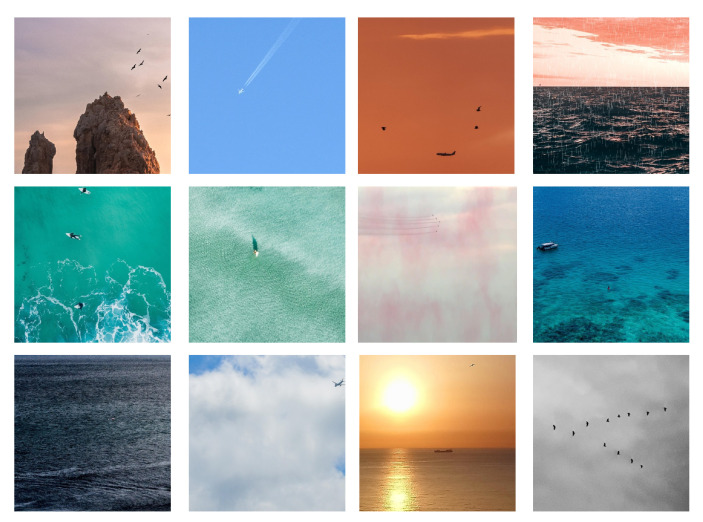
Example images from the self-constructed visible-light small target dataset.

To address the scarcity of annotated data, we construct a dataset containing 843 visible-light images with small targets. An improved CycleGAN model is then employed to translate these visible-light images into the infrared domain, generating pseudo-infrared images to augment the training data for infrared small target detection tasks. The targets in our custom dataset include aircraft, ships, birds, humans, masts, parachutes, hot air balloons, buoys, and surfboards. The backgrounds feature diverse scenes such as the ocean, urban environments, clouds, sky, rivers, and mountains. Compared to publicly available datasets, our dataset includes a wider range of target categories and more complex, diverse background scenarios. Example images are shown in Figure 2.

To generate high-quality pseudo-infrared images, we modify the original CycleGAN architecture in three key aspects. First, we replace both the generator and discriminator backbones with a U-Net architecture. The encoder–decoder framework and skip connections of U-Net preserve spatial details and resolution, which is especially beneficial for infrared scenes containing small targets or strong background noise. Second, we incorporate the CBAM module. This attention mechanism enables the network to focus automatically on discriminative channels and spatial regions, thereby amplifying the response of small targets while suppressing background interference. Third, to improve the geometric consistency and semantic integrity of target regions in the pseudo-infrared images, we add an IoU loss term to the original adversarial loss and cycle-consistency loss of CycleGAN. By leveraging the available target annotations, this IoU loss provides explicit regional supervision, guiding the generator to maintain the location, contour, and semantic features of targets while performing style translation. This addition alleviates target semantic shifts that arise from the different physical characteristics of the two modalities. We define the overall loss function for the visible-to-infrared style translation task using the improved CycleGAN model as follows:

(1) $$L_{total}=L_{GAN}^{G_{A\to B}}+L_{GAN}^{G_{B\to A}}+\lambda L_{cycle}+L_{IoU}$$

where λ is a hyperparameter used to balance the importance of the cycle-consistency loss. When the weight is too small, the cycle-consistency constraint becomes insufficient, potentially causing semantic drift in the generated images. On the other hand, if the weight is too large, it may interfere with adversarial learning, reducing the quality of the generated images. Therefore, based on empirical experience, λ is set to 5.

$L_{GAN}^{G_{A\to B}}$, $L_{GAN}^{G_{B\to A}}$, $L_{cycle}$, and $L_{IoU}$ denote the adversarial loss functions for the generators $G_{A\to B}$ and $G_{B\to A}$, the cycle-consistency loss, and the IoU loss, respectively. The loss for the generator $G_{A\to B}$ is formulated as follows:

(2) $$L_{\mathrm{GAN}}^{G_{A\to B}}=-E_{x∼P_{X}}\left[\log D_{B}\left(G_{A\to B}\left(x\right)\right)\right]$$

where $x∼P_{X}$ denotes a sample drawn from the visible-light domain X. The generator $G_{A\to B}$ aims to map visible-light images to the infrared domain, producing synthetic infrared images $G_{A\to B}\left(x\right)$. The discriminator $D_{B}$ is trained to distinguish between real infrared images and generated ones. It outputs a probability in [0,1], indicating the likelihood that an input image is real. By maximizing $\log D_{B}\left(G_{A\to B}\left(x\right)\right)$, the generator is encouraged to produce outputs that are statistically indistinguishable from real infrared images, thereby improving the fidelity of domain translation. Since the loss is defined with a negative sign, minimizing it encourages the generator to produce more realistic images.

Similarly, the loss for the reverse mapping is defined as follows:

(3) $$L_{GAN}^{G_{B\to A}}=-E_{y∼P_{Y}}\left[\log D_{A}\left(G_{B\to A}\left(y\right)\right)\right]$$

where $y∼P_{Y}$ denotes a real sample from the target domain Y, and $G_{B\to A}$ is the generator mapping target images back to the source domain.

However, above two losses cannot ensure semantic consistency between input and output images. Therefore, we introduce a cycle consistency loss defined as follows:

(4) $$L_{\mathrm{cycle}}=E_{x∼P_{X}}\left[{∥G_{B\to A}\left(G_{A\to B}\left(x\right)\right)-x∥}_{1}\right]+E_{y∼P_{Y}}\left[{∥G_{A\to B}\left(G_{B\to A}\left(y\right)\right)-y∥}_{1}\right]$$

where this loss contains two terms: one ensures that a source-domain image mapped to the target domain and then back $G_{B\to A}\left(G_{A\to B}\left(x\right)\right)$ closely resembles the original input *x*. And the other ensures the same for *y* from the target domain. Where ${∥\cdot ∥}_{1}$ denotes the $\ell_{1}$ norm, which measures pixel-wise differences and helps preserve structural and edge details.

(5) $$L_{IoU}=1-\frac{\left|Y^{\prime }\cap Y\right|}{\left|Y^{\prime }\right|+\left|Y\right|-\left|Y^{\prime }\cap Y\right|}$$

In Equation (5), $Y^{\prime }\cap Y$ denotes the intersection between the predicted mask $Y^{\prime }$ and the ground-truth mask *Y*, and $|Y^{\prime }\cap Y|$ represents the number of pixels in this intersection.

**Figure 3:**
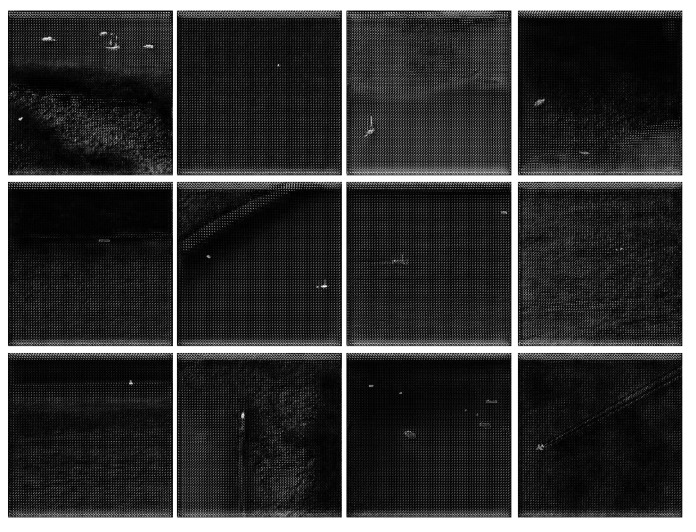
Examples of the generated pseudo-infrared small target images.

As a result, the improved CycleGAN not only possesses strong style adaptation capabilities but also preserves critical structural information of small targets in infrared images more accurately, providing more reliable pseudo-sample support for subsequent semi-supervised detection tasks. The generated pseudo-infrared images more closely resemble real infrared images and exhibit better adaptability in target detection tasks. Examples of the generated pseudo-infrared small target images are shown in Figure 3.

### 3.3. Wavelet-Enhanced Channel Recalibration and Fusion (WECRF) Module

We design an efficient and lightweight wavelet-enhanced channel recalibration and fusion (WECRF) module by integrating channel and spatial attention, channel weight recalibration, and feature fusion mechanisms. This module helps the model perform more precise segmentation of targets. The detailed structure of the module is shown in Figure 1.

After entering this module, the input features *X* first undergo a wavelet transformation, producing one low-frequency component and three high-frequency components, each with half the size of the original feature map. These four components are then concatenated to form a new feature map with a reduced spatial size and a channel dimension four times larger than the original.

(6) $$\left\{X^{LL},\;X^{LH},\;X^{HL},\;X^{HH}\right\}=\mathrm{WT}\left(X\right).$$

(7) $$F=C\;\left[X^{LL},\;X^{LH},\;X^{HL},\;X^{HH}\right]\in R^{4C\times \frac{H}{2}\times \frac{W}{2}},$$

where WT denotes the wavelet transform, and C denotes the concatenation operation.

To address this, a channel weight recalibration operation is applied. Specifically, the channels of the input tensor are first divided into G groups. The channels are then reordered so that channels from different groups are interleaved.

(8) $$\tilde{F}=R_{G}\left(F\right).$$

(9) $$\hat{F}=\phi \;\left(\mathrm{BN}(Conv(\tilde{F}))\right).$$

where $R_{G}$ denotes the channel group reordered operation, ϕ denotes the ReLU function. After interleaving, the channel dimension is flattened, and a new tensor is returned with recalibrated channel arrangements. The main purpose of this operation is to promote inter-channel interaction and enhance feature mixing by rearranging the channel order. This is motivated by the observation that in deep neural networks, each channel in a feature map typically captures specific attributes, such as edges, textures, or semantic content. In grouped convolutions or other grouped operations, channels within different groups remain independent, which limits information exchange and may lead to overly homogeneous features. By allowing information to be exchanged across groups through channel interleaving, the model gains richer feature representations and improved expressive capability.

**Figure 4:**
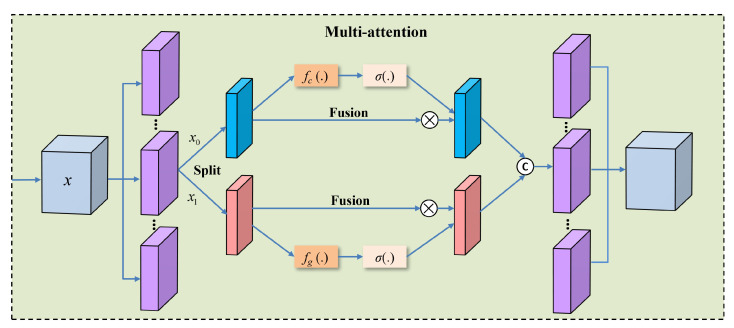
Structural diagram of the multi-attention module.

After that, we combine channel attention and spatial attention mechanisms to construct multi-attention components to enhance feature representations. Spatial attention, on the other hand, calculates the correlation of local spatial context using group normalization, further enhancing the feature representation. By combining these two attention mechanisms, the network’s ability to focus on important regions is significantly improved, enabling better capture of local details in infrared small targets. As shown in Figure 4, the multi-attention module first reshapes the input feature map *x* by grouping its channels, resulting in multiple sub-feature maps. Each group is then split into two parts ($x_{0}$ and $x_{1}$) to compute channel attention $c_{g}$ and spatial attention $s_{g}$ separately.

(10) $${\hat{F}}_{g}=\left[x_{0,g},\;x_{1,g}\right],\;g=1,\ldots ,G.$$

For channel attention, global average pooling is first applied to $x_{0}$ to obtain global information for each group. This global information is then passed through a linear transformation $f_{c}\left(\cdot \right)$, implemented with learnable weights and biases, to calculate the importance of each channel. A sigmoid activation function σ(·) is used to generate the channel attention map, which is subsequently applied to $x_{0}$ to reweight the channel features.

(11) $$c_{g}=\sigma \;\left(f_{c}\left(\mathrm{GAP}\left(x_{0,g}\right)\right)\right)$$

(12) $$x_{0,g}^{\prime }=c_{g}⊙x_{0,g}$$

When computing spatial attention, group normalization is first applied to $x_{1}$ to extract spatial attention information. This is then adjusted using weights and biases, followed by a sigmoid activation function to generate the spatial attention map. The spatial attention is applied to $x_{1}$ to refine the spatial features. Finally, the attention-weighted feature maps $x_{0}$ and $x_{1}$ are concatenated to form a new feature map.

(13) $$s_{g}=\sigma \;\left(f_{s}\left(\mathrm{GN}\left(x_{1,g}\right)\right)\right)$$

(14) $$x_{1,g}^{\prime }=s_{g}⊙x_{1,g}$$

Finally, the attention-weighted feature maps $x_{0}$ and $x_{1}$ are concatenated to form a new feature map:

(15) $${\hat{F}}_{g}^{\prime }=C\;\left[x_{0,g}^{\prime },\;x_{1,g}^{\prime }\right],$$

To further exploit the relationship between channels and spatial locations, we perform an additional channel weight recalibration after the hybrid attention module. Because channels are interrelated and the features of different wavelet components complement one another at global and local scales, simple concatenation cannot capture these dependencies effectively. Moreover, target responses to high-frequency features vary across spatial positions. To address this, we group the channels and then reorder them, thus breaking the isolation of features within a single wavelet component (for example, low-frequency or high-frequency bands). After reordering, each channel contains information from multiple wavelet components, enabling cross-dimensional interaction among components. Although spatial attention has already refined the spatial distribution of features, it does not eliminate the separation of features within channels. Channel reordering promotes the cross-channel fusion of spatial information, further uncovering the intrinsic connections between channel and spatial domains. This operation mitigates feature isolation, strengthens inter-channel interaction, and reduces feature redundancy, thereby enhancing the feature-representation capability of the WRSSNet model.

### 3.4. Loss Function

Our semi-supervised loss consists of three components: the loss $L_{tru}$ computed from real infrared images, the loss $L_{pse}$ computed from pseudo-infrared images, and the consistency loss $L_{con}$ derived from unlabeled infrared images. The definitions of these three loss terms are as follows:

(16) $$L_{tru}=L_{IoU}+L_{BCE}$$

(17) $$L_{pse}=L_{tru}$$

(18) $$L_{con}=L_{MSE}$$

In the above equations, $L_{IoU}$, $L_{BCE}$, and $L_{MSE}$ represent the IoU loss, BCE loss, and MSE loss, respectively. Based on this, our total loss is defined as follows:

(19) $$L_{all}=L_{tru}+\alpha L_{pse}+\beta L_{con}$$

where α and β are weighting hyperparameters, which are empirically initialized to 0.5 and 0.1, respectively.

The BCE loss is suitable for pixel-wise binary segmentation tasks (e.g., distinguishing targets from the background in infrared target detection). As a convex function, it is theoretically easy to optimize. However, when used alone, it may be biased towards the background if the target occupies a very small portion of the image. Therefore, we combine it with IOU loss to address class imbalance. In semi-supervised or multi-task learning, MSE is often used to assess the consistency between pseudo-labels and ground truths, providing a consistency constraint for the model. This loss is more sensitive to deviations between predictions and targets, making it suitable for tasks requiring precise alignment. It also avoids gradient explosion issues when predictions approach boundary values (e.g., 0 or 1), and it integrates well with other loss functions. Therefore, we adopt a combination of IOU loss, BCE loss, and MSE loss as our total loss. The definitions of the BCE and MSE losses are as follows:

(20) $$L_{BCE}=-\frac{1}{N}\sum_{i=1}^{N}\left[y_{i}\log\left(x_{i}\right)+\left(1-y_{i}\right)\log\left(1-x_{i}\right)\right]$$

(21) $$L_{MSE}=\frac{1}{N}\sum_{i=1}^{N}{\left(x_{i}-y_{i}\right)}^{2}$$

where *N* denotes the number of samples, $x_{i}$ is the model-predicted output, and $y_{i}$ is the target label. In the context of semi-supervised learning, they correspond to the pseudo-label predicted by the model and the re-predicted pseudo-label after data augmentation, respectively.

## 4. Experiment

### 4.1. Experimental Data and Evaluation Metrics

#### 4.1.1. Datasets Description

NUAA (Nanjing University of Aeronautics and Astronautics, Nanjing, China)-SIRST [4] is a public dataset provided by Nanjing University of Aeronautics and Astronautics (NUAA), specifically designed for single-frame infrared small target detection. It contains a total of 427 infrared images, each annotated with one or more target regions. The target shapes are relatively regular, primarily appearing as point-like or small blob-like regions. The infrared images are collected from real-world scenes under various weather and noise conditions, including environments such as mountains, clouds, sky, and ocean. IRSTD-1K [34], constructed by the team at Northwestern Polytechnical University (NWPU), comprises diverse scenes and target types. This dataset consists of 1000 infrared images with a uniform resolution of 512 × 512 pixels. NUDT (National University of Defense Technology, Changsha, China)-SIRST [32] is a public dataset for infrared small target detection, released by the National University of Defense Technology (NUDT). It contains 1327 images of uniform size, 256 × 256 pixels. The infrared images are captured from a variety of real-world scenarios, including ground-based, aerial, and maritime backgrounds. Each image contains one or more small targets with varying shapes and sizes, such as point-like targets, ellipses, and ship contours. Many targets exhibit motion blur or low contrast, and the dataset includes complex environmental conditions such as dense cloud cover and ocean wave reflections. The NUAA-SIRST and IRSTD-1K datasets are collected from real infrared images, while the NUDT-SIRST dataset is synthetic. We use the real infrared images from the NUAA-SIRST dataset to train the discriminator of the improved CycleGAN, resulting in pseudo-infrared images. In addition, our method leverages both infrared images from each public dataset and synthetically generated pseudo-infrared images for training, respectively. Specifically, infrared images with pixel-level annotations provide accurate supervision for the network, while pseudo-infrared images generated from visible-light images are used to augment the training data and enhance the model’s generalization ability under diverse background conditions. In our experiments, the above three public datasets are divided into three parts: a test set, a labeled training set, and an unlabeled training set.

#### 4.1.2. Evaluation Metrics

We use the target detection rate $\left(P_{d}\right)$, false alarm rate $\left(F_{a}\right)$, Intersection over Union (IoU), normalized Intersection over Union (nIoU), and F1 score (F1) as the evaluation metrics for our experiments. They are defined as follows:

(22) $$P_{d}=\frac{N_{pred}}{N_{all}}$$

(23) $$F_{a}=\frac{N_{false}}{N_{all-p}}$$

where $N_{pred}$ is the number of true targets that are correctly detected, and $N_{all}$ is the number of all the true targets, $N_{false}$ is the number of false pixel detections, and $N_{all-p}$ is the number of all image pixels.

(24) $$IoU=\frac{TP}{T+P-TP}$$

(25) $$nIoU=\frac{1}{N}\sum_{i}^{N}\frac{TP(i)}{T(i)+P(i)-TP(i)}$$

(26) $$F_{1}=\frac{2TP}{2TP+FP+FN}$$

where *N* is the total number of samples, and *T* and *P* represent the number of true and predicted positive pixels, respectively. *TP, FP*, and *FN* represent the number of true positive, false positive, and false negative pixels, respectively.

### 4.2. Implementation Details

To validate the effectiveness of the proposed WRSSNet, we conducted infrared small target detection experiments on the NUAA-SIRST, IRSTD-1K and NUDT-SIRST datasets. Our method was compared with several state-of-the-art approaches, including UIU-Net, AGPCNet, DNA-Net, and UCFNet. Our model was implemented using Python 3.8 and PyTorch 1.13.1. For the style transfer model, we adopted the Adam optimizer with a learning rate of 0.0002, a minimum learning rate of $10^{-7}$, a weight decay of $10^{-2}$, and exponential decay rates of 0.5 and 0.999. We used the ReduceLROnPlateau scheduler and trained the model for a total of 300 epochs with a batch size of 2. For the wavelet-attention-based semi-supervised model, we employed the AdamW optimizer with a learning rate of 0.01, a weight decay of $10^{-4}$, and decay rates of 0.9 and 0.999. The model was trained for 500 epochs on the NUAA dataset and 300 epochs on the IRSTD-1K and NUDT-SIRST datasets, with a batch size of 4. All experiments were conducted on a system equipped with an NVIDIA RTX 4090 GPU (24 GB VRAM), an AMD Ryzen 9 5900X CPU, 64 GB of DDR4 memory, and NVMe SSD storage.

### 4.3. Quantitative Results and Analysis

To improve pseudo-infrared image synthesis beyond previous CGAN-based approaches [29], we introduce two key enhancements. First, we incorporate an IoU-based loss alongside the standard adversarial and cycle-consistency losses to better preserve structural consistency and object shapes, enhancing spatial localization. Second, we replace the IR-GAN generator and discriminator with a U-Net architecture and embed a convolutional block attention module, which, together with skip connections, preserves fine details of small targets. As shown is Table 1, compared with the improved CGAN, our method achieves consistent gains in SSIM, MS-SSIM, and FSIM, providing high-quality synthetic data for semi-supervised infrared small target detection.

**Figure 5:**
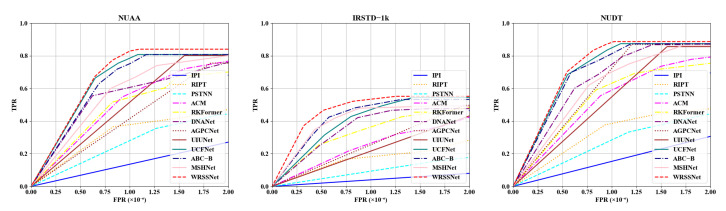
The ROC curves of different methods on the NUAA-SIRST, IRSTD-1K, and NUDT-SIRST datasets. Our WRSSNet achieves the highest $P_{d}$ at a very low $F_{a}$.

To more objectively demonstrate the superiority of our method, we present the evaluation metric results of various methods on the NUAA-SIRST, IRSTD-1K, and NUDT-SIRST datasets, respectively. As shown, our method achieves outstanding performance on the NUAA-SIRST dataset, with an IoU of 69.89 and an nIoU of 70.10, significantly outperforming other methods. The results on the NUDT-SIRST dataset show that our method achieves a Pd = 88.68%, Fa = 10.21, IoU = 74.34%, nIoU = 73.02%, and F1 = 86.15%, significantly outperforming existing methods. Compared to NUAA-SIRST and NUDT-SIRST, the overall scores on the IRSTD-1K dataset are lower due to stronger background clutter, lower contrast, and smaller target sizes. Nevertheless, our method still outperforms other competing methods on the IRSTD-1K dataset, as detailed in Table 2, Table 3 and Table 4. In addition, Figure 5 presents the ROC curves of all methods on the three datasets. The ROC curve of WRSSNet consistently stays closest to the top-left corner, clearly demonstrating its ability to achieve high detection rates while maintaining a low false alarm rate.

As shown in Table 2, our method achieved the best performance on all metrics except for the detection rate, where the result is still close to the highest value. This indicates that our method performs excellently on the NUAA-SIRST dataset.

As shown in Table 3, we further compared the performance of our method with three other approaches on the IRSTD-1K dataset. Due to the characteristics of this dataset, all methods exhibited relatively poor performance in the semi-supervised infrared small target detection task. Nevertheless, our method still outperformed the others, indicating its effectiveness. At the same time, these results also suggest that there is still room for improvement in our approach.

### 4.4. Visualization Results and Analysis

#### 4.4.1. Dataset Construction

**Figure 6:**
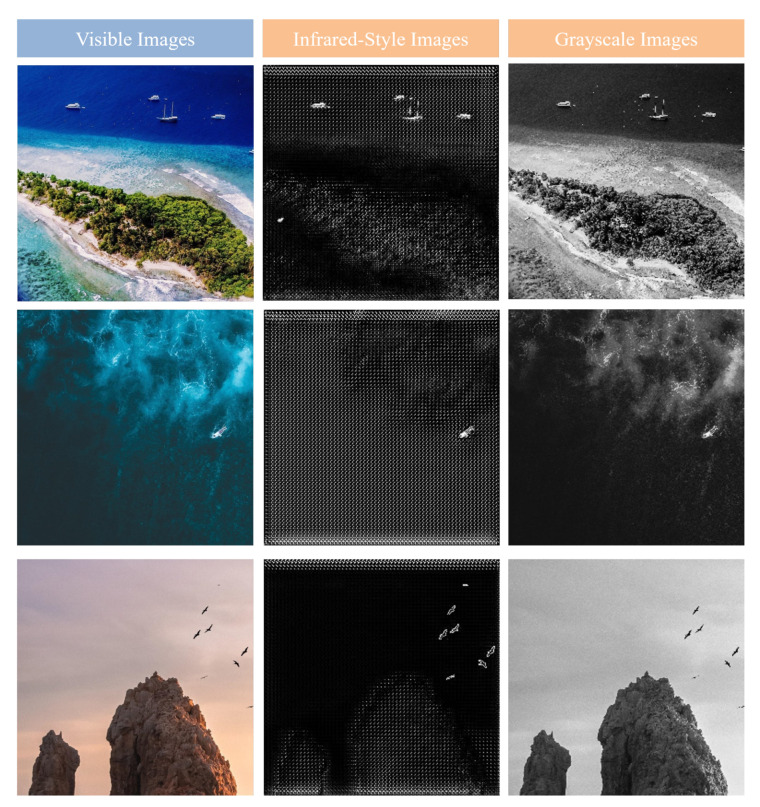
Comparison Between Visible Images, Infrared-Style Images, and Grayscale Images.

Figure 6 shows the results of converting visible images into infrared-style images using our method, as well as the results of directly converting visible images into grayscale. It can be seen that, compared to the original visible and grayscale images, the infrared-style images contain target and background information that more closely resembles real infrared images. Therefore, infrared-style images can effectively augment infrared data.

#### 4.4.2. Small Object Detection

**Figure 7:**
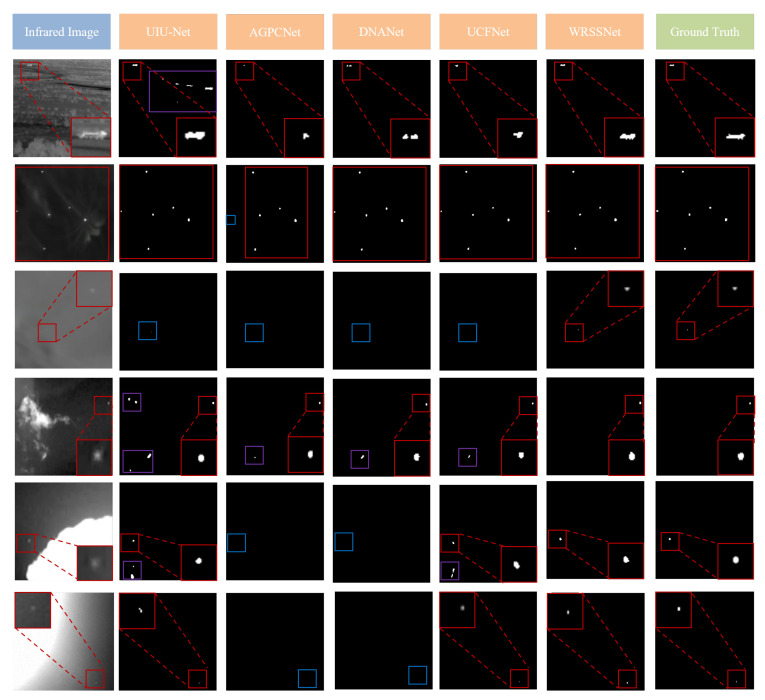
Visualization results of different methods. Color frames indicate detection results: red denotes true-positive detections, blue denotes missed targets, and purple denotes false-positive detections.

Figure 7 presents six selected target images along with the detection results obtained by our method and several comparative methods. In these visualizations, red bounding boxes indicate ground-truth targets, purple boxes denote false detections, and blue boxes represent missed targets. The first row of Figure 7 shows that most methods can accurately detect relatively bright targets with regular shapes. The second row demonstrates that, except for AGPCNet, most methods can also effectively handle multiple targets in simple backgrounds. The third and second-to-last rows illustrate that the comparative methods perform poorly when detecting dim, low-contrast small targets. The fourth row indicates that some comparative methods lack robustness against noise, often misidentifying noise as targets. In contrast, our method consistently delivers strong performance across these challenging scenarios.

**Figure 8:**
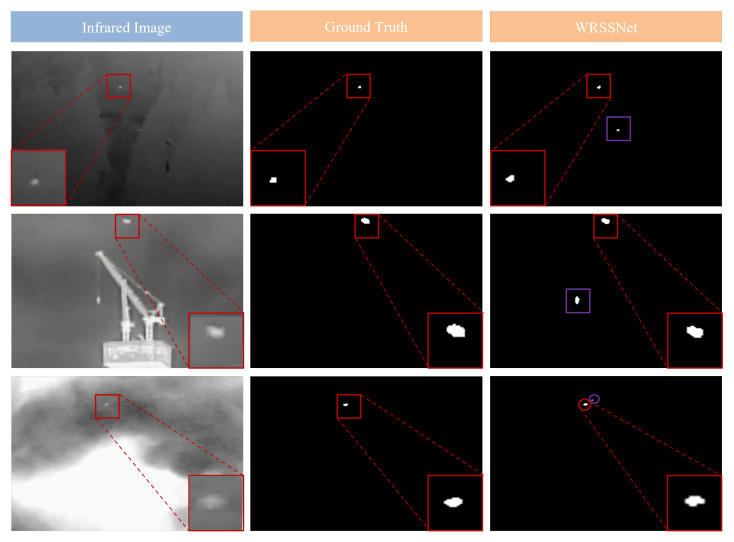
Examples of false positives produced by the model. Red frames and circles denote true-positive detections, purple frames and circles denote false-positive detections.

**Figure 9:**
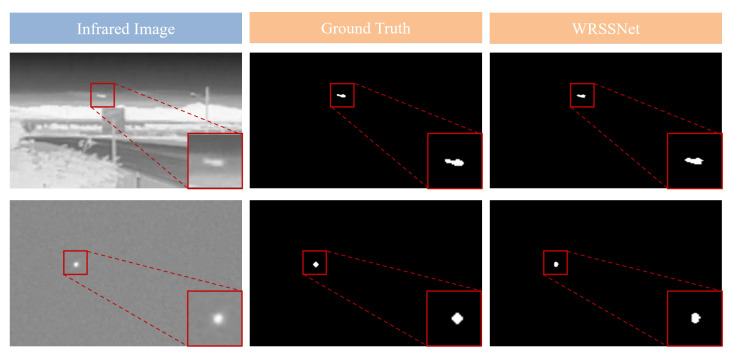
Examples of target deformation in model predictions.

Although our method outperforms the four compared methods in detecting multiple targets, handling low-contrast images, and resisting noise interference, it is not without limitations. Certain failure cases still occur in some test images. Figure 8 and Figure 9 show examples of detection results where our model performs suboptimally. As shown in Figure 8, although the proposed method is capable of accurately detecting small targets in infrared images with multiple noise points, low contrast, and dim brightness, it still occasionally misidentifies noise as small targets. This misclassification often occurs because the noise shares a similar brightness, structure, or size with the actual targets. To address this issue, future work may focus on generating more training samples with noisy targets through data augmentation, incorporating more powerful denoising modules, or enhancing the noise suppression mechanisms. These improvements could help the model reduce false detections caused by noise to a certain extent.

As shown in Figure 9, the proposed method effectively detects small targets in infrared images with heavy noise and low contrast. However, some detected targets exhibit shape deformation or partial loss. In the first row, the detected target appears to be missing a portion in the lower right compared to the ground truth. In the second row, the target in the original image has a diamond-like shape, while the detected result is closer to a circular shape, indicating deformation. These issues suggest that the method’s ability to capture edge information of targets still needs improvement.

### 4.5. Ablation Experiments

A series of ablation experiments are conducted on the NUAA-SIRST and IRSTD-1K datasets to evaluate the effectiveness of the style transfer (ST) and the wavelet-enhanced channel recalibration and fusion (WECRF) module. The ablation results for ST and WECRF are shown in Table 5. It can be observed that both ST and WECRF improve the performance of the first three metrics based on the UCFNet baseline. When used together, they achieve the best results on these metrics, indicating a certain degree of complementarity between the two. The decline in the Pd metric may be related to the quality of the generated infrared-style images, which we plan to further improve in future work.

To justify the choice of the Daubechies-4 (db4) wavelet, we provide theoretical analysis and ablation results. Db4’s four vanishing moments preserve localized intensity changes that are typical of infrared small targets while suppressing large-scale background structures, unlike Haar. Compared with Coiflets of the same order, db4 has shorter support, reducing computation and memory usage. Ablation experiments in Table 6 confirm that db4 achieves higher detection accuracy and lower false-alarm rates with minimal overhead, making it the most suitable wavelet for our task.

While DNANet and UIU-Net employed attention and multi-scale fusion in the spatial domain through simple concatenation, WECRF explicitly separates features in the frequency domain via wavelet decomposition and incorporates channel reordering and recalibration to enhance interactions between high- and low-frequency components, and between salient and non-salient regions. This design more effectively preserves edges and fine details of weak infrared small targets. Ablation experiments on NUAA-SIRST show that integrating WECRF into the baseline improves IoU, F1, and Pd while reducing Fa, demonstrating benefits beyond conventional attention mechanisms. The ablation experiment results are shown in Table 7.

### 4.6. Sensitivity Analysis

The hyperparameters α and β in Equation (19) are used to balance two loss terms. The first is computed from pseudo-infrared images ($L_{pse}$), and the second is the consistency loss ($L_{con}$) derived from unlabeled infrared images during the semi-supervised training stage. We conduct a grid-search-based sensitivity analysis for different combinations of α and β. To balance computational cost and statistical significance, we adopt a step size of 0.2, resulting in 25 grid search configurations. As shown in Table 8, WRSSNet maintains high performance across a broad parameter range, with α=0.5 and β=0.1 achieving the optimal trade-off between the detection rate (Pd) and false alarm rate (Fa).

## 5. Conclusions

To address the limitations caused by data scarcity, we propose a semi-supervised infrared small target detection method based on wavelet recalibration. The scarcity of annotated data makes it difficult for models to learn weak features, capture multi-scale characteristics, and distinguish targets from complex backgrounds. To alleviate the lack of labeled data, we first construct a visible-light small target dataset. Then, we use an improved CycleGAN model to convert it into pseudo-infrared images, which are used to expand the training set. To make use of unlabeled infrared data, we propose a semi-supervised network named WRSSNet. This model incorporates a wavelet-enhanced channel recalibration and fusion (WECRF) module. The module strengthens the interaction between channel and spatial features. It also improves multi-scale feature representation by reweighting and fusing features effectively. We conduct extensive experiments on several public infrared small target datasets. The results confirm the effectiveness of WRSSNet. Our method provides a flexible and generalizable solution for infrared small target detection. While WRSSNet excels in multi-target detection, low-contrast scenarios, and noise robustness, it still faces challenges. Bright noise points resembling real targets may cause false alarms, and target shapes may appear as deformed due to imperfect boundary capture. In future work, we will focus on enhancing the quality of infrared images, reducing noise in pseudo-infrared data, and further improving denoising performance as well as boundary detail preservation.

## Figures and Tables

**Table 1:** Comparison experiments on the quality of pseudo-infrared images generated with the improved CGAN. Uparrow indicates higher is better. Downarrow indicates lower is better. Best results are in bold.

Method	SNR↑	PSNR↑	RMSE↓	SSIM↑	MS-SSIM↑	FSIM↑
Improved CGAN [29]	18.7	**22.4**	7.9	0.818	0.805	0.829
Our method	**19.4**	21.9	**6.8**	**0.855**	**0.837**	**0.855**

**Table 2:** Quantitative evaluation on the NUAA-SIRST dataset in IoU (%), nIoU (%), F1 (%), AUC, Pd (%), and Fa $\left(10^{-6}\right)$. Best results are in bold and second-best are underlined.

Methods	IoU	nIoU	F1	AUC	Pd	Fa
IPI [2]	27.64	23.74	53.12	0.461	68.11	158.95
RIPT [10]	18.38	15.91	44.21	0.448	68.05	35.56
PSTNN [11]	31.52	31.92	48.00	0.420	68.08	39.72
ACM [4]	61.03	59.17	72.31	0.570	81.39	26.36
RKFormer [3]	63.89	61.23	73.75	0.531	73.10	25.45
DNANet [32]	65.32	64.68	78.56	0.549	77.62	21.04
AGPCNet [8]	65.41	61.77	76.36	0.561	75.54	18.22
UIUNet [9]	64.48	60.92	75.72	0.590	80.22	15.57
ABC-B [19]	65.50	64.18	78.58	0.618	80.73	11.67
MSHNet [12]	62.34	61.85	75.26	0.558	80.20	20.11
UCFNet [20]	67.69	65.00	78.79	0.639	80.77	**10.83**
WRSSNet (ours)	**70.10**	**69.89**	**82.28**	**0.663**	**84.05**	10.97

**Table 3:** Quantitative evaluation on the IRSTD-1K dataset in IoU (%), nIoU (%), F1 (%), AUC, Pd (%), and Fa $\left(10^{-6}\right)$. Best results are in bold and second-best are underlined.

Methods	IoU	nIoU	F1	AUC	Pd	Fa
IPI [2]	14.98	13.51	26.05	0.280	46.37	348.18
RIPT [10]	17.33	11.43	20.35	0.289	44.48	37.66
PSTNN [11]	15.93	13.71	27.48	0.267	40.34	59.15
ACM [4]	35.21	34.03	55.38	0.340	48.91	23.21
RKFormer [3]	38.82	38.08	56.13	0.355	49.16	21.22
DNANet [32]	38.32	37.06	52.47	0.348	47.65	16.02
AGPCNet [8]	39.75	38.37	56.21	0.380	49.63	19.47
UIUNet [9]	36.28	35.96	56.16	0.372	48.77	22.60
ABC-B [19]	42.52	41.59	60.58	0.426	53.25	13.12
MSHNet [12]	39.37	38.29	56.07	0.404	49.24	16.47
UCFNet [20]	41.50	40.23	59.33	0.402	54.46	14.31
WRSSNet (ours)	**44.42**	**42.18**	**61.51**	**0.468**	**55.17**	**12.57**

**Table 4:** Quantitative evaluation on the NUDT-SIRST dataset in IoU (%), nIoU (%), F1 (%), AUC, Pd (%), and Fa $\left(10^{-6}\right)$.

Methods	IoU	nIoU	F1	AUC	Pd	Fa
IPI [2]	29.10	24.85	54.70	0.473	69.50	145.21
RIPT [10]	19.20	16.50	45.36	0.447	67.81	33.14
PSTNN [11]	32.67	32.95	49.25	0.417	69.24	36.41
ACM [4]	61.83	60.50	73.97	0.576	82.70	24.84
RKFormer [3]	62.00	60.85	75.10	0.573	78.17	23.98
DNANet [32]	70.55	68.12	84.90	0.613	86.80	14.12
AGPCNet [8]	71.20	69.77	85.12	0.608	86.95	12.03
UIUNet [9]	69.81	68.36	84.10	0.592	85.60	15.61
ABC-B [19]	72.05	70.62	85.85	0.692	87.33	11.95
MSHNet [12]	70.17	69.65	84.60	0.600	86.57	17.35
UCFNet [20]	72.11	71.68	85.71	0.703	87.42	10.90
WRSSNet (ours)	**74.34**	**73.02**	**86.15**	**0.722**	**88.68**	**10.21**

**Table 5:** Ablation study results of different components on IoU (%), nIoU (%), Pd (%), F1 (%), and Fa $\left(10^{-6}\right)$.

Method	IoU	nIoU	Pd	F1	Fa
UCFNet	67.69	65.00	80.77	78.79	**10.83**
UCFNet + WECRF	68.73	68.37	81.84	80.73	10.91
UCFNet + ST	68.42	67.35	81.96	80.78	10.94
UCFNet + ST + WECRF	**70.10**	**69.89**	**84.05**	**82.28**	10.97

**Table 6:** Ablation experiments with Haar and Coiflets-4.

Wavelet	IoU	nIoU	Pd	F1	Fa	Params (M)
Haar	67.02	66.75	81.10	79.39	24.61	**3.25**
Coiflets-4	69.12	68.84	83.70	81.80	12.03	3.42
Daubechies-4 (db4)	**70.10**	**69.89**	**84.05**	**82.28**	**10.97**	3.30

**Table 7:** Ablation experiments of embedding CSAM and our WECRF into the baseline.

Method	IoU	nIoU	Pd	F1	Fa
baseline	67.69	65.00	80.77	78.79	**10.83**
baseline + CSAM	68.06	67.67	81.34	79.58	11.51
baseline + WECRF	**68.73**	**68.37**	**81.84**	**80.73**	10.91

**Table 8:** Sensitivity analysis of hyperparameters α and β.

α	β	IoU	nIoU	F1	Pd	$F_{a}$
0.1	0.1	67.30	67.12	80.25	81.40	12.78
0.1	0.3	66.85	66.68	79.90	80.95	13.15
0.1	0.5	66.40	66.23	79.54	80.50	13.52
0.1	0.7	65.95	65.78	79.18	80.05	13.89
0.1	0.9	65.50	65.33	78.82	79.60	14.26
0.3	0.1	68.75	68.58	81.50	82.95	11.60
0.3	0.3	68.30	68.13	81.15	82.50	11.97
0.3	0.5	67.85	67.68	80.80	82.05	12.34
0.3	0.7	67.40	67.23	80.45	81.60	12.71
0.3	0.9	66.95	66.78	80.10	81.15	13.08
**0.5**	**0.1**	**70.10**	**69.89**	**82.28**	**84.05**	**10.97**
0.5	0.3	69.65	69.44	81.93	83.60	11.34
0.5	0.5	69.20	68.99	81.58	83.15	11.71
0.5	0.7	68.75	68.54	81.23	82.70	12.08
0.5	0.9	68.30	68.09	80.88	82.25	12.45
0.7	0.1	69.85	69.65	82.05	83.82	11.05
0.7	0.3	69.40	69.20	81.70	83.37	11.42
0.7	0.5	68.95	68.75	81.35	82.92	11.79
0.7	0.7	68.50	68.30	81.00	82.47	12.16
0.7	0.9	68.05	67.85	80.65	82.02	12.53
0.9	0.1	69.60	69.40	81.82	83.59	11.18
0.9	0.3	69.15	68.95	81.47	83.14	11.55
0.9	0.5	68.70	68.50	81.12	82.69	11.92
0.9	0.7	68.25	68.05	80.77	82.24	12.29
0.9	0.9	67.80	67.60	80.42	81.79	12.66

## Data Availability

The data presented in this study are available on request from the corresponding author, Jingwen Ma.
